# Extracellular calcium increases bisphosphonate-induced growth inhibition of breast cancer cells

**DOI:** 10.1186/bcr1845

**Published:** 2008-01-11

**Authors:** Fabrice Journé, Naïma Kheddoumi, Carole Chaboteaux, Hugues Duvillier, Guy Laurent, Jean-Jacques Body

**Affiliations:** 1 Laboratory of Endocrinology and Bone Diseases, Institut Jules Bordet, Université Libre de Bruxelles, rue Héger-Bordet, B-1000, Brussels, Belgium; 2 Laboratory of Experimental Hematology, Institut Jules Bordet, Université Libre de Bruxelles, rue Héger-Bordet, B-1000, Brussels, Belgium; 3 Laboratory of Histology, Faculty of Medicine and Pharmacy, University of Mons-Hainaut, avenue du Champ de Mars, B-7000, Mons, Belgium

## Abstract

**Introduction:**

Bisphosphonates have become standard therapy for the treatment of skeletal complications related to breast cancer. Although their therapeutic effects mainly result from an inhibition of osteoclastic bone resorption, *in vitro *data indicate that they also act directly on breast cancer cells, inhibiting proliferation and inducing apoptosis.

**Methods:**

The present study examined the effects of calcium (from 0.6 to 2.0 mmol/l) on the antitumour activity of the bisphosphonate ibandronate (1 to 1,000 nmol/l) on MDA-MB-231 and MCF-7 breast cancer cells. Cell culture densities were determined using crystal violet staining assay. Apoptotic cell death was assessed by annexin V-phycoerythrin and 7-amino-actinomycin double staining.

**Results:**

At low calcium concentration, 30 μmol/l ibandronate had no effect on MDA-MB-231 cells growth and only slightly inhibited MCF-7 cells growth. Higher calcium levels significantly increased growth inhibition as well as cell apoptosis induced by ibandronate. We observed similar effects with zoledronic acid. Of note, enhancement of ibandronate-induced growth inhibition was also observed in other breast cancer cell lines (T-47D, ZR-75, Hs-578T and BT-549 cells). The growth inhibitory effect of ibandronate in the presence of high concentrations of calcium was partly suppressed by the calcium chelator EGTA (ethylene glycol tetra-acetic acid). In addition, in the presence of calcium at high concentrations, cells accumulated more [^14^C]ibandronate than at low calcium concentrations. We obtained further evidence of enhancement of cellular ibandronate accumulation by calcium by demonstrating that high calcium levels increased the inhibition of protein prenylation induced by the bisphosphonate.

**Conclusion:**

Altogether, our data suggest that extracellular calcium, probably through its binding to ibandronate, markedly increased its cellular accumulation and its inhibitory activity on breast tumour cells. Thus, calcium released during the process of tumour-induced osteolysis might enhance the antitumour effects of bisphosphonates and contribute to their therapeutic efficacy.

## Introduction

Breast carcinoma is the most frequent malignancy occurring in women of Western developed countries. The skeleton is the commonest site for metastasis of breast cancer. Bone metastases represent a major public health concern because of their frequency and the severe morbidity that they generate in cancer patients (hypercalcaemia, severe pain, pathological fractures and so on) [1].

Bisphosphonates are used as the standard therapy for hypercalcaemia complicating breast cancer and they are now commonly administered for the treatment of bone metastases. These potent agents inhibit the activity of osteoclasts and induce their apoptosis [2]. In addition to their effects of bone cells, recent *in vitro *and animal studies have shown that the bisphosphonates also directly inhibit the proliferation and induce the apoptosis of osteotropic cancer cells [3-8]. By inhibiting osteoclast-mediated bone resorption, they can also interrupt the vicious circle between tumour cells, osteoclasts and bone matrix, thereby limiting the release of growth factors. Moreover, they can directly inhibit the mitogenic effect of growth factors on cancer cells [9].

Bisphosphonates are nonhydrolyzable analogues of pyrophosphate. They are effective chelators of calcium [10], they have high affinity for the bone tissue and they preferentially accumulate at sites of active bone remodelling [11]. Previous data [12] indicated that a fluorescent analogue of bisphosphonate is rapidly internalized into intracellular vesicles in osteoclasts. Fluid-phase endocytosis is involved in the initial internalization of bisphosphonates into vesicles, and endosomal acidification is then required for the exit of bisphosphonate from vesicles and entry into cytosol.

There are two classes of bisphoshonates that differ with regard to structure and mechanism of action [13]. The first one includes pyrophosphate-resembling bisphosphonates, such as clodronate and etidronate, which are metabolically incorporated into nonhydrolyzable ATP analogues that act as inhibitors of ATP-dependent enzymes [14]. The second class is more recent and includes nitrogen-containing bisphosphonates, such as alendronate, pamidronate, risedronate, ibandronate and zoledronic acid, which interfere with the mevalonate pathway, inhibiting the farnesyl diphosphate synthase and blocking the farnesylation and the geranylgeranylation of small GTPase proteins [15]. In the case of nitrogen-containing bisphosphonates, inhibition of protein prenylation is probably the main mechanism of action that leads to cytotoxic effects, even though additional mechanisms have been advanced [16]. In particular, a previous study [17] reported that alendronate increased intracellular calcium concentration in osteoclasts. Under some circumstances, an increase in intracellular calcium level may cause cell death by necrosis or apoptosis [18].

The skeleton is a repository for mineral, especially calcium. Consequently, tumour-induced bone resorption leads to the release of large quantities of calcium into the bone microenvironment. Interestingly, the local calcium level at resorption sites has been reported to rise as high as 40 mmol/l [19]. Hence, metastatic breast cancer cells near resorbing osteoclasts are likely to be exposed to 'hypercalcaemic' conditions (2.0 mmol/l), which may influence therapeutic efficacy.

In spite of considerable therapeutic progress achieved with the use of bisphosphonates, the current treatment protocols reduce bone morbidity by 'only' 40% to 50%, suggesting that these drugs block tumour-induced osteolysis only partially [20]. A better understanding of the mechanisms of action of bisphosphonates on tumour cells would make it possible to use them more efficiently and to improve clinical results. In the present study we examined the effects of an increase in extracellular calcium level (0.6 to 2.0 mmol/l) on the antitumour activity of ibandronate in MDA-MB-231 and MCF-7 breast cancer cell lines.

## Materials and methods

### Breast cancer cell lines and culture conditions

The MCF-7 (HTB-22), MDA-MB-231 (HTB-26), T-47D (HTB-133), ZR-75-1 (CRL-1500), Hs 578T (HTB-126) and BT-549 (HTB-122) breast cancer cell lines were obtained from the American Type Culture Collection (Manassas, VA, USA). Cells were cultured at 37°C in a humidified 95% air and 5% carbon dioxide atmosphere. For routine maintenance, cells were propagated in 75 cm^2 ^flasks containing complete RPMI medium consisting of RPMI 1640 with phenol red, supplemented with 5% heat-inactivated foetal bovine serum and with L-glutamine, penicillin and streptomycin at standard concentrations (all from Gibco BRL, Life Technologies, Merelbeke, Belgium). Cells were harvested by trypsinization (0.1% trypsin, 0.02% EDTA) and subcultured twice weekly.

For experiments, cells were plated in complete RPMI medium. One day after seeding, the culture medium was replaced by fresh medium adjusted to the final desired calcium concentrations (from 0.6 to 2.0 mmol/l) by adding CaCl_2_.2H_2_O (Sigma, St Louis, MO, USA) along with contain drugs and/or other chemicals. Of note, standard RPMI medium contains 0.6 mmol/l calcium, as calculated from the manufacturer's data. Ibandronate and [^14^C]ibandronate (102 mCi/mmol) were supplied by Roche Diagnosics GmbH (Penzberg, Germany) and F. Hoffmann-La Roche Ltd (Basel, Switzerland), respectively. Zoledronic acid was supplied by Novartis (Basel, Switzerland). Paclitaxel, ethylene glycol tetra-acetic acid (EGTA) and MgCl_2 _were from Sigma. Calcium-sensing receptor (CaR) agonist (NPS R-467) and antagonist were kindly provided by Dr J Fox (NPS Pharmaceuticals, Salt Lake City, UT, USA). Cells were treated for 1 to 72 hours, as specified in Results (see below).

### Cell growth assay

Cell number was assessed indirectly by staining with crystal violet dye, as described in a previous report [21]. Briefly, cells were seeded in 96-well plates at a density of 2,500 cells/well in complete RPMI medium and cultured for 24 hours. Cells were then exposed for 72 hours or less (pulse exposures) to calcium, drugs and other compound(s) alone or in combination, as described in Results (see below). Medium was removed, cells were gently rinsed with phosphate-buffered saline (PBS), fixed with 1% glutaraldehyde/PBS for 15 minutes and stained with 0.1% crystal violet (weight/vol in double distilled H_2_O) for 30 minutes. Cells were destained under running tap water for 15 minutes and subsequently lysed with 0.2% Triton X-100 (vol/vol in double distilled H_2_O). The absorbance was measured at 550 nm using a Microplate Autoreader EL309 (BIO-TEK Instruments, Winooski, VT, USA). Blank wells containing medium alone were used for background subtraction and sham-treated cells were cultured in parallel as controls.

### Apoptosis determination

Apoptotic cell death was assessed using annexin V-phycoerythrin (PE) apoptosis detection kit I (BD Pharmingen, Erembodegem, Belgium), in accordance with the manufacturer's recommendations. Briefly, cells were seeded in six-well plates (density 50,000 cells/well), cultured for 24 hours and treated with bisphosphonates in presence or absence of additional calcium concentrations for 72 hours, as described in Results (see below). Cell monolayers were washed twice in PBS, harvested by trypsinization, centrifuged and resuspended in 100 μl 1× Binding Buffer (BD Pharmingen). After addition of 5 μl annexin V-PE and 5 μl of 7-amino-actinomycin (7-AAD, a vital dye), cell suspensions were incubated for 15 minutes at room temperature in darkness. Finally, cell samples were diluted with 400 μl 1× binding buffer and analyzed in a flow cytometer (FACSCalibur, Becton Dickinson, Franklin Lakes, NJ, USA) within 1 hour. Data are presented as percentages of annexin V-PE positive and 7-AAD negative cells.

### Western blot analysis

Unprenylated Rap1A and total Rap1 were determined by Western blotting. Cells were plated at a density of 10^4 ^cells/cm^2 ^in 60 cm^2 ^Petri dishes containing complete RPMI medium, cultured for 24 hours and then incubated for 24 additional hours with calcium (0.6 or 2.0 mmol/l) and ibandronate (1 to 1,000 μmol/l) or vehicle as specified in Results (see below). Cell monolayers were rinsed twice with Tris-buffered saline (50 mmol/l Tris-HCl [pH 7.5], 150 mmol/l NaCl), lysed in Tris-buffered saline containing 0.5% sodium deoxycholate, 1% Nonidet P-40, 0.1% SDS, 50 mmol/l NaF, 0.1 mM Na_3_VO_4 _and 5 mmol/l EDTA with freshly added proteolysis inhibitors, and finally centrifuged. Protein concentrations in cell lysate supernatants were determined by the BCA Protein Assay (Pierce, Rockford, IL, USA) using bovine serum albumin as standard. Equal amounts of protein were subjected to Western blotting using a goat polyclonal anti-human Rap1A antibody (SC-1482; Santa Cruz Biotechnology, Santa Cruz, CA, USA) diluted 1:1,000, which recognizes the unprenylated form of the small GTPase Rap1A [22], and a rabbit polyclonal anti-human Rap1 antibody (SC-65; Santa Cruz Biotechnology) diluted 1:500. Peroxidase-labelled anti-goat IgG antibody (1:1,500; Pierce) and peroxidase-labelled anti-rabbit IgG antibody (1:2,500; Amersham Pharmacia Biotech, Roosendaal, The Netherlands) were used as secondary reagents to detect corresponding primary antibodies. Bound peroxidase activity was revealed using the SuperSignal^® ^West Pico Chemiluminescent Substrate (Pierce). Immunostaining signals were digitalized with a PC-driven LAS-3000 CCD camera (Fujifilm, Tokyo, Japan), using software specifically designed for image acquisition (Image Reader, Raytest^®^, Straubenhardt, Germany).

### Measurement of [^14^C]ibandronate accumulation in cells

Bisphosphonate accumulation by breast cancer cells was determined by using [^14^C]ibandronate. Cells were seeded in 12-well plates at a density of 40,000 cells/well in complete RPMI medium and cultured for 24 hours. Cells were then incubated in fresh medium supplemented or not supplemented with calcium and containing 10 μmol/l [^14^C]ibandronate (102 mCi/mmol) for 4 hours, as described in Results (seee below). Cell monolayers were rinsed and lysed as described under Western blot analysis (see above). The amounts of [^14^C]ibandronate associated with cell lysate supernatants were determined using a liquid scintillation counter (Wallac 1409; PerkinElmer, Waltham, MA, USA). Protein concentrations in the same samples were measured by the BCA Protein Assay (Pierce), using bovine serum albumin as standard. Results were expressed as picomoles of ibandronate per milligram of protein.

### Statistical analysis

Data are reported as means ± standard deviation, and statistical analysis was performed by analysis of variance. Dunnett *post hoc *test was used to compare treated conditions with the untreated condition (control), and Tukey *post hoc *test was performed for multiple comparisons between groups. The level of statistical significance was arbitrarily set at 0.01. All analyses were conducted using SPSS software (SPSS Inc., Paris, France).

## Results

### High extracellular calcium concentration increases ibandronate-induced cell growth inhibition and cell apoptosis

Breast cancer cells were cultured in complete RPMI medium containing 5% foetal bovine serum and supplemented or not supplemented with CaCl_2_, to achieve calcium concentrations from 0.6 to 2.0 mmol/l. Cell growth was assessed after 72 hours of treatment with ibandronate.

**Figure 1:**
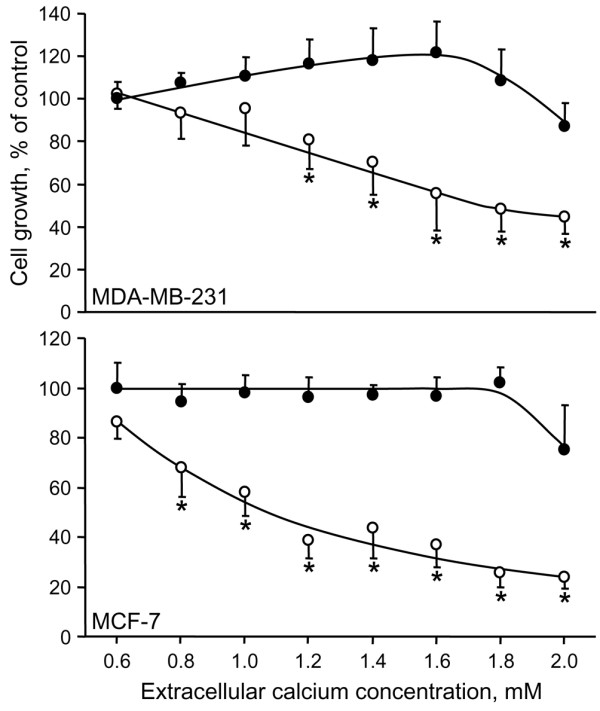
Effect of calcium on the growth inhibition induced by ibandronate in MDA-MB-231 and MCF-7 cells. Cells were treated for 72 hours with 30 μmol/l ibandronate (open circles) in the presence of increasing concentrations of calcium (0.6 to 2.0 mmol/l; filled circles, no ibandronate). Cell culture densities were determined by crystal violet staining assay. Data are expressed as percentages (mean ± standard deviation) of control values, which refer to cultures in presence of 0.6 mmol/l calcium and without ibandronate. Mean of results pooled from three experiments (*n *= 18). *P < 0.01 versus no ibandronate, analysis of variance, Tukey *post hoc *test.

**Figure 2:**
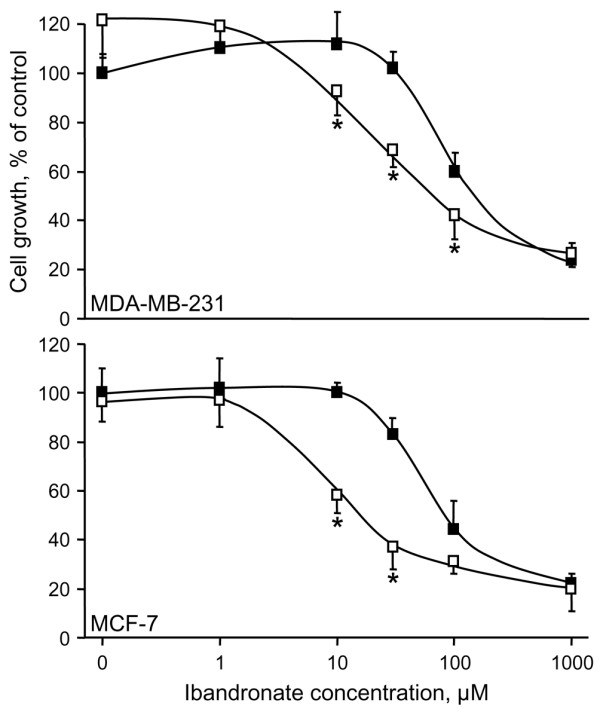
Concentration-effect relationships of ibandronate on cancer cell proliferation in hypocalcaemic or hypercalcaemic conditions. MDA-MB-231 and MCF-7 cells were exposed for 72 hours to increasing ibandronate concentrations (1 to 1,000 μmol/l) in culture medium containing 0.6 mmol/l calcium (filled squares) or 1.6 mmol/l calcium (open squares). Cell culture densities were evaluated by crystal violet staining assay. Data are presented as percentages (mean ± standard deviation) of control values, which refer to cultures with 0.6 mmol/l calcium and without ibandronate. Mean of results pooled from three experiments (*n *= 18). *P < 0.01 versus 0.6 mmol/l calcium, analysis of variance, Tukey *post hoc *test.

In the presence of 0.6 mmol/l calcium, 30 μmol/l ibandronate had no effect on MDA-MB-231 cell growth, whereas it slightly inhibited MCF-7 cell growth by 13.6 ± 6.6% (Figure 1). Higher extracellular calcium concentrations enhanced the inhibitory effects of ibandronate in a dose-dependent manner. Indeed, in the presence of 2.0 mmol/l calcium, 30 μmol/l ibandronate dramatically inhibited cell proliferation by 55.5 ± 7.8% and 76.1 ± 4.6% (*P *< 0.01) in MDA-MB-231 and MCF-7 cells, respectively. Half maximal inhibitory concentration (IC_50_) values for growth inhibition by ibandronate were determined in presence of 0.6 and 1.6 mmol/l calcium. Dose-response curves revealed that an increase in calcium concentration decreased the IC_50 _values of ibandronate from 150 to 60 μmol/l in MDA-MB-231 cells and from 80 to 10 μmol/l in MCF-7 cells (Figure 2 and Table 1). Similar effects were also documented in four additional breast cancer cell lines (T-47D, ZR-75-1, Hs-578T and BT-549; Table 1), indicating that these observations are not restricted to particular cell lines. Of note, a higher sensitivity of tumour cells to ibandronate appeared to be associated with the oestrogen receptor (ER) status, but appeared to be independent of the type of bone lesions that the tumour cells may develop in nude mice. Altogether, these data indicated that high calcium concentration enhanced the growth inhibitory potency of ibandronate in breast cancer cells.

**Table 1:** Comparison of the effect of ibandronate on six breast cancer cell lines

			IC_50 _(μmol/l)	
			
Cell line	ER status^a^	Bone lesions^b^	0.6 mmol/l calcium	1.6 mmol/l calcium
T-47D	+	Blastic	35^c^	2
ZR-75-1	+	Lytic	50	15
MCF-7	+	Blastic	80	10
Hs-578T	-	Blastic	100	10
BT-549	-	Lytic	150	10
MDA-MB-231	-	Lytic	150	60

^a^As described by American Type Culture Collection (Manassas, VA, USA). ^b^As defined by Yin and coworkers [32] and Lacroix and colleagues [33]. ^c^Half maximal inhibitory concentration (IC_50_) values determined from concentration-effect curves for cancer cells exposed for 72 hours to ibandronate (1 to 1,000 μmol/l) in the presence of 0.6 or 1.6 mmol/l calcium. Cell culture densities were evaluated by crystal violet staining assay. Means of results pooled from three experiments (*n *= 18). ER, oestrogen receptor.

**Figure 3:**
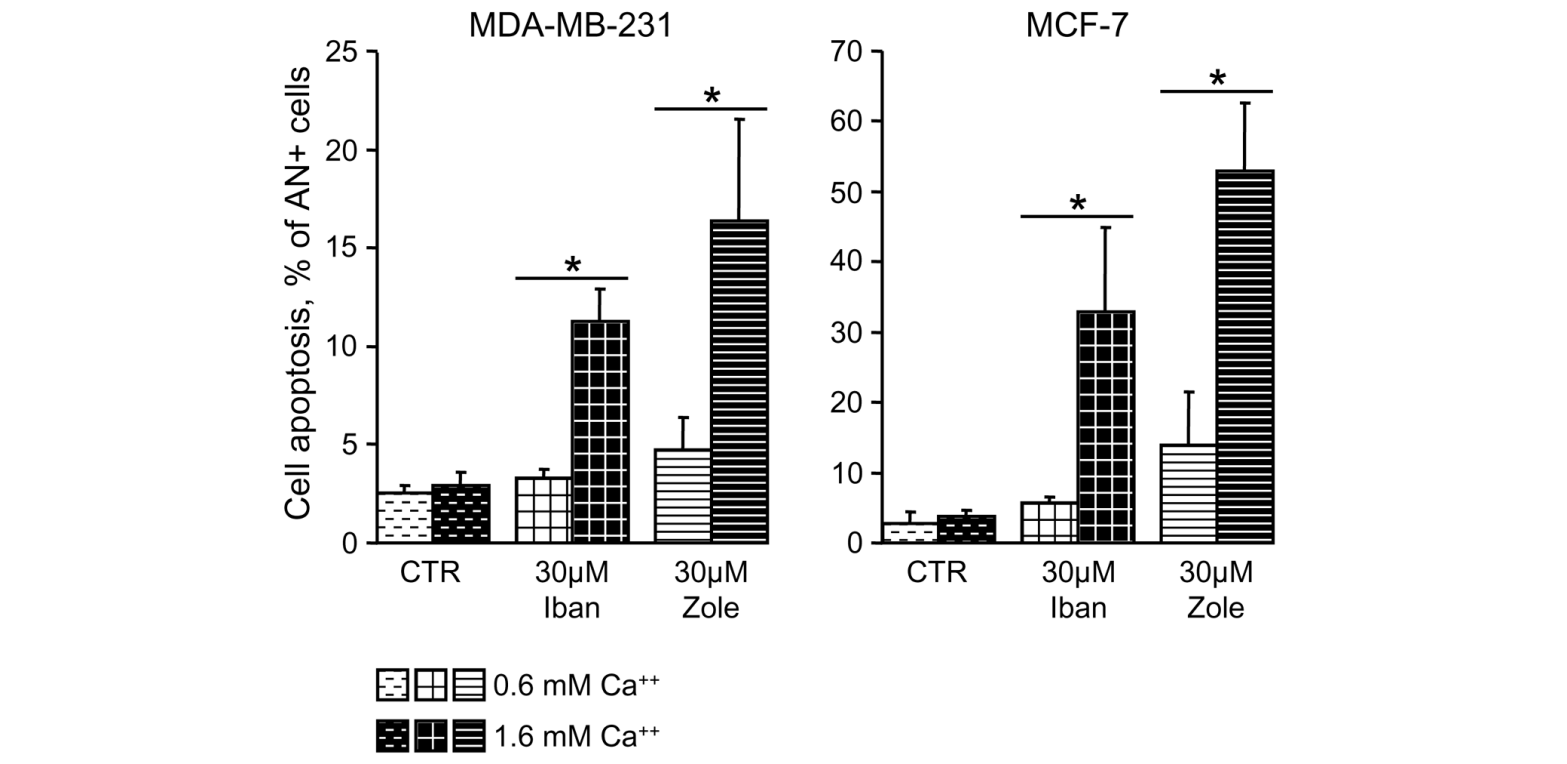
Effect of calcium on cell apoptosis induced by bisphosphonates in MDA-MB-231 and MCF-7 cells. Cells were incubated for 72 hours with 30 μmol/l ibandronate (Iban) or zoledronic acid (Zole) in culture medium containing 0.6 or 1.6 mmol/l calcium. Apoptotic cell death was assessed by annexin V-phycoerythrin (PE) and 7-amino-actinomycin (7-AAD) double staining. Data are presented as percentages (mean ± standard deviation) of annexin V-PE-positive (AN^+^) and 7-AAD-negative cells. Mean of results pooled from two experiments (*n *= 4). *P < 0.01 versus 0.6 mmol/l calcium, analysis of variance, Tukey *post hoc *test.

Annexin V-PE labelling was used to detect apoptotic cell death. Cell apoptosis rates were low (<3% in both cell lines) in the control condition (0.6 mmol/l calcium) and did not significantly change in presence of 1.6 mmol/l calcium (Figure 3). Ibandronate (30 μmol/l) did not induce significant cell apoptosis in presence of 0.6 mmol/l calcium. By contrast, it significantly increased the percentage of annexin V-PE-positive cells in the presence of 1.6 mmol/l calcium (11.4% and 32.9% in MDA-MB-231 and MCF-7 cells, respectively; Figure 3). Similar observations were obtained with zoledronic acid, except that the latter drug provoked more extensive apoptosis, particularly in presence of 1.6 mmol/l calcium (17% and 52% for MDA-MB-231 and MCF-7 cells, respectively).

**Figure 4:**
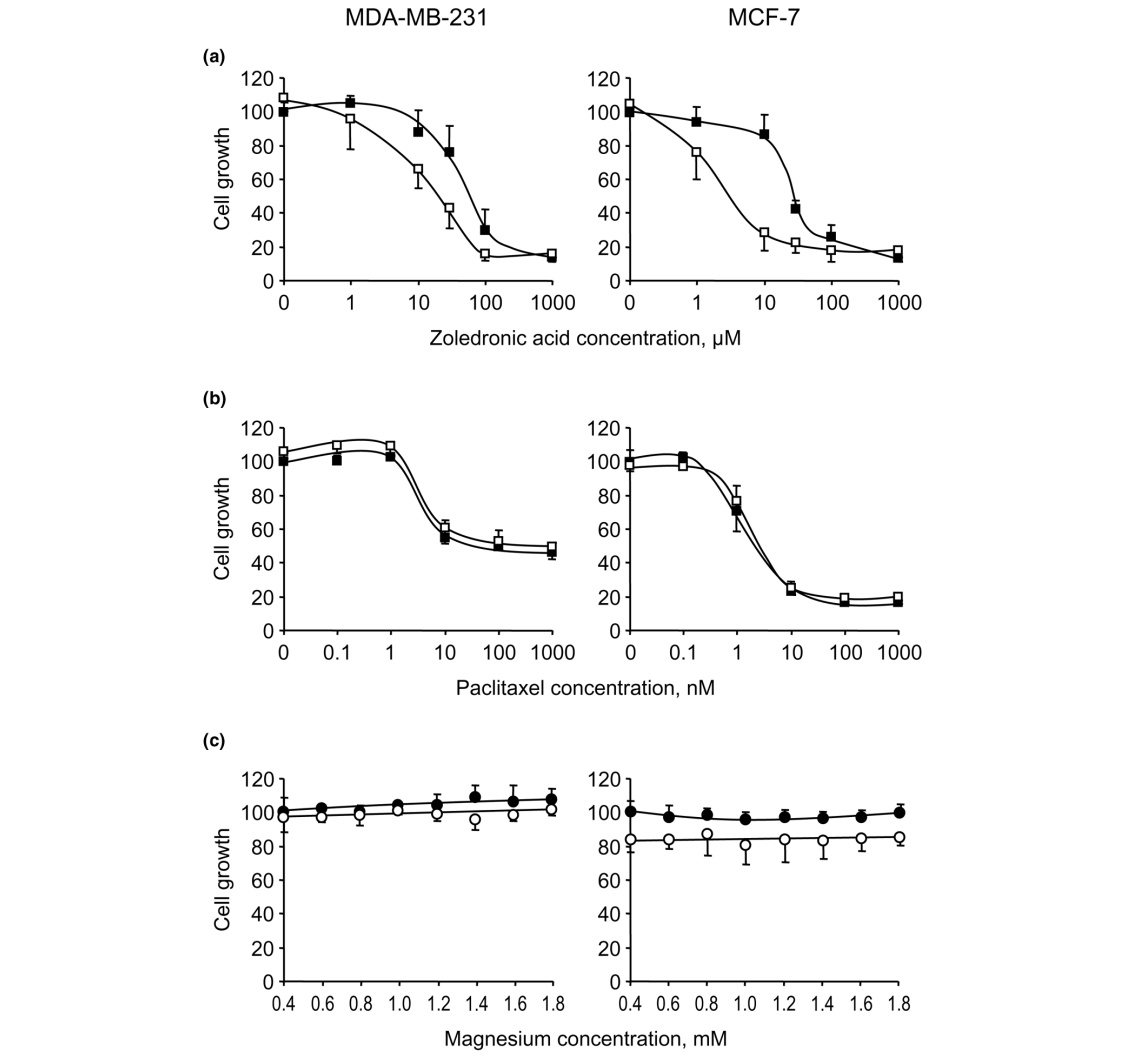
Comparative study of calcium effect on zoledronic acid or paclitaxel, and magnesium effect on ibandronate. Cells were exposed for **(a) **72 hours to increasing concentrations of zoledronic acid (1 to 1,000 μmol/l) or **(b) **to increasing concentrations of paclitaxel (0.1 to 1,000 μmol/l), in culture medium containing 0.6 mmol/l calcium (filled squares) or 1.6 mmol/l calcium (open squares), or **(c) **to 30 μmol/l ibandronate (open circles) in the presence of increasing magnesium concentrations (0.4 to 1.8 mmol/l; filled circles, no ibandronate). Cell culture densities were measured by crystal violet staining assay. Data are presented as percentages (mean ± standard deviation) of control values, which refer to culture with 0.6 mmol/l calcium or 0.4 mmol/l magnesium and without drug. Mean of results pooled from two experiments (*n *= 12).

**Figure 5:**
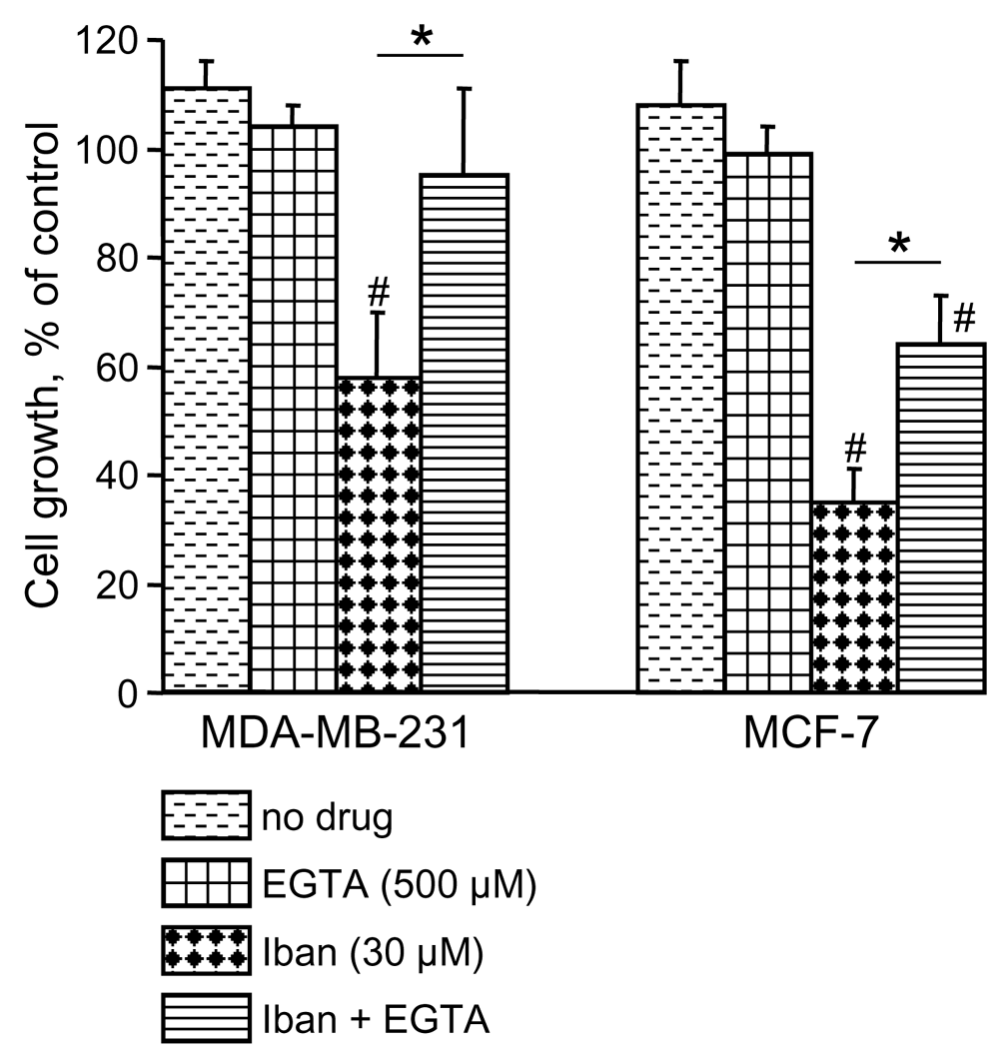
Effect of EGTA on growth inhibition induced by ibandronate in presence of high calcium concentration. MDA-MB-231 and MCF-7 cells were treated for 72 hours with 30 μmol/l ibandronate (Iban) alone or with 500 μmol/l ethylene glycol tetra-acetic acid (EGTA) in culture medium containing 1.6 mmol/l calcium. Cell culture densities were determined by crystal violet staining assay. Data are presented as percentages (mean ± standard deviation) of control values, which refer to culture with 1.6 mmol/l calcium and without drugs. Mean of results pooled from two experiments (*n *= 12). *P < 0.01 between indicated conditions, analysis of variance; ^#^*P *< 0.01 versus control, analysis of variance; Tukey *post hoc *test.

As documented above by analyzing bisphosphonate-induced apoptosis, the modulating effect of calcium was not restricted to ibandronate, because this cation also increased the growth inhibition produced by zoledronic acid (Figure 4a). Thus, dose-response curves showed that an increase in calcium level from 0.6 to 1.6 mmol/l diminished the IC_50 _values of zoledronic acid from 50 to 15 μmol/l in MDA-MB-231 cells and from 20 to 2 μmol/l in MCF-7 cells. Interestingly, these effects were specific to bisphosphonates because calcium had no effect on the cytotoxicity induced by the structurally unrelated antimitotic agent paclitaxel (Figure 4b). In addition, the enhancement of ibandronate activity was specifically noted with calcium, inasmuch as it was not observed with the other divalent cation magnesium in the same range of concentrations (Figure 4c). On the other hand, calcium chelation by EGTA at 0.5 mmol/l, a concentration that did not affect cell growth, significantly reduced the growth inhibitory effect induced by 30 μmol/l ibandronate in culture medium containing 1.6 mmol/l calcium (Figure 5). Similarly, calcium chelation by 100 μmol/l clodronate, a pyrophosphate-resembling bisphosphonate that had no detectable effect on cell survival at this concentration, significantly reversed the growth inhibition induced by ibandronate (data not shown).

### Calcium-sensing receptor is not involved in the effect of calcium on ibandronate-induced cell growth inhibition

**Figure 6:**
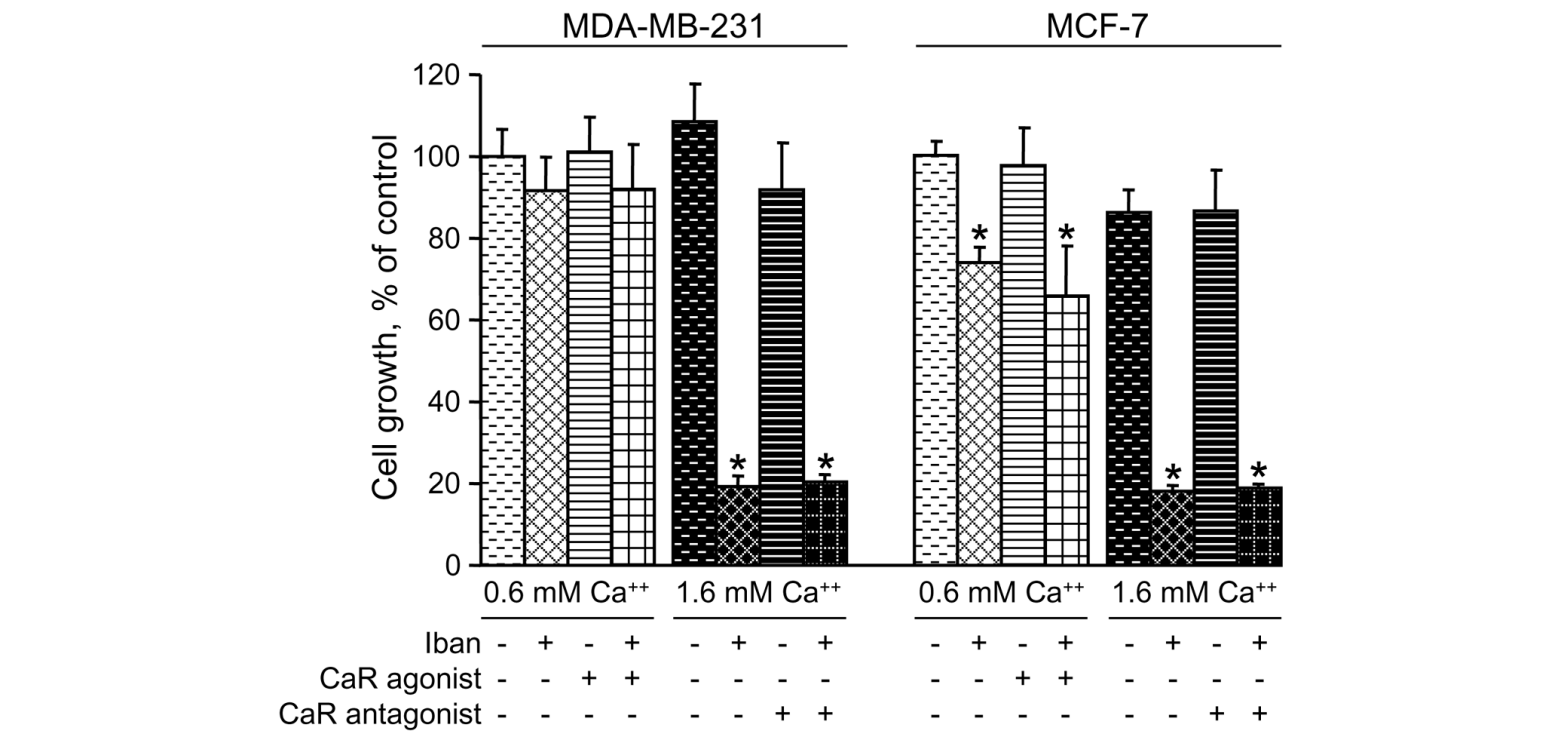
Effects of CaR agonist and antagonist on growth inhibition caused by ibandronate in cancer cells. MDA-MB-231 and MCF-7 cells were incubated for 72 hours with 30 μmol/l ibandronate (Iban) and/or calcium-sensing receptor (CaR) agonist in culture medium containing 0.6 mmol/l calcium, or with 30 μmol/l ibandronate and/or CaR antagonist in culture medium containing 1.6 mmol/l calcium. Cell culture densities were evaluated by crystal violet staining assay. Data are presented as percentages (mean ± standard deviation) of control values, which refer to culture with 0.6 mmol/l calcium and without drugs. Mean of results pooled from two experiments (*n *= 12). *P < 0.01 versus control, analysis of variance, Dunnett *post hoc *test.

A potential role of the CaR in mediating the effects of calcium on cell growth inhibition induced by ibandronate was checked by using CaR regulators. The CaR agonist NPS R-467 at 10^-5 ^mol/l did not mimic the effects of high extracellular calcium concentrations, because it did not enhance growth inhibition by ibandronate (Figure 6). Likewise, the CaR antagonist at 10^-6 ^mol/l did not change the growth inhibitory effect of ibandronate at 1.6 mmol/l calcium concentration. Therefore, these data excluded the possibility that CaR might be involved in the modulation of bisphosphonate effect by calcium.

### High extracellular calcium concentration enhances [^14^C]ibandronate accumulation in breast carcinoma cells and increases ibandronate effect on prenylation

**Figure 7:**
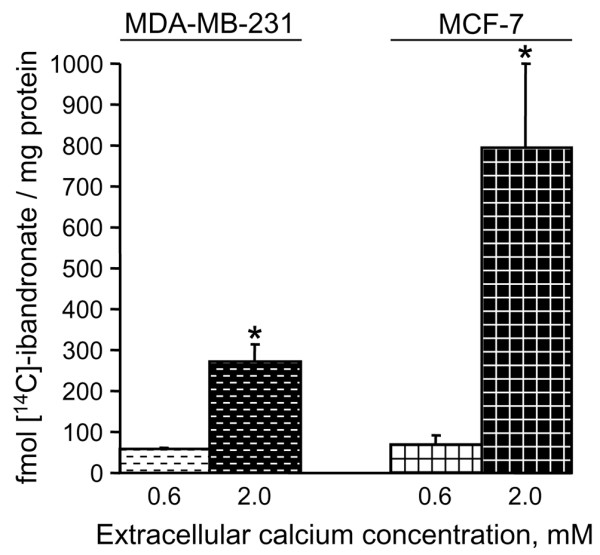
Effect of calcium on [^14^C]ibandronate accumulation in MDA-MB-231 and MCF-7 cells. Cells were exposed for 4 hours to 10 μmol/l [^14^C]ibandronate (102 mCi/mmol) in culture medium containing 0.6 or 2.0 mmol/l calcium. The quantities of [^14^C]ibandronate associated with cell lysate supernatants were measured using a liquid scintillation counter. Results are presented as percentages (mean ± standard deviation) of control values, which refer to culture with 0.6 mmol/l calcium. Mean of results performed in triplicate. *P < 0.01 versus control, analysis of variance, Dunnett *post hoc *test.

In order to address the effects of calcium on cellular accumulation of ibandronate, MDA-MB-231 and MCF-7 cells were cultured in medium containing 0.6 or 2.0 mmol/l calcium and were exposed to 10 μmol/l [^14^C]ibandronate for 4 hours (Figure 7). In the presence of 2.0 mmol/l calcium, cells accumulated more radiolabelled ibandronate than cells cultured in presence of 0.6 mmol/l calcium (about 4.6-fold and 11.4-fold increases in MDA-MB-231 and MCF-7 cells, respectively). Interestingly, MCF-7 cells accumulated threefold more [^14^C]ibandronate than did MDA-MB-231 cells, which could contribute to the higher sensitivity of the former cells to bisphosphonates (Figure 1 to 3).

**Figure 8:**
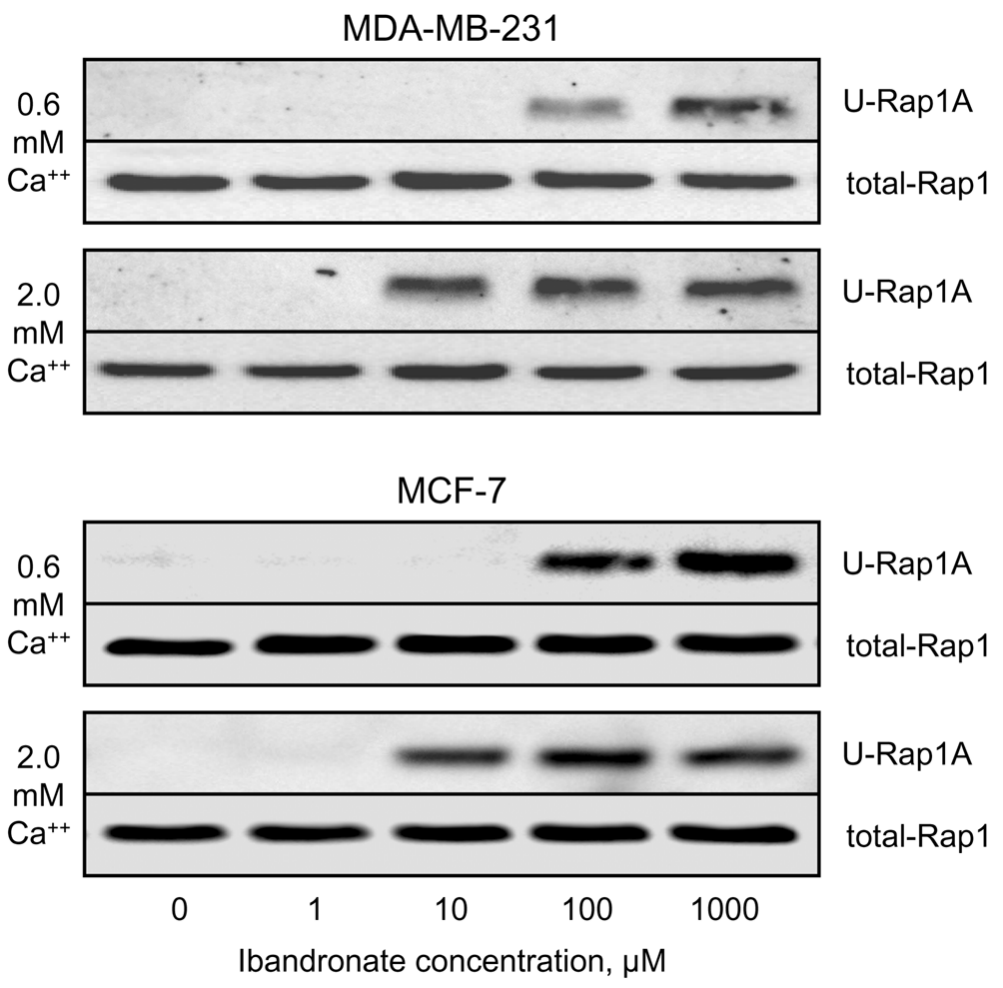
Effect of calcium on ibandronate-induced inhibition of Rap1A prenylation in MDA-MB-231 and MCF-7 cells. Cells were incubated for 24 hours with increasing concentrations of ibandronate (1 to 1,000 μmol/l) in culture medium containing 0.6 or 2.0 mmol/l calcium, lysed and subjected to Western blot analysis. Equal amounts of proteins (25 μg) were subjected to 12% SDS-PAGE and electrotransferred onto nitrocellulose membranes. Immunodetection was performed with a goat polyclonal anti-human antibody raised against the unprenylated form of Rap1A. Total Rap1 protein (input) was evaluated in parallel using a rabbit polyclonal anti-human Rap1 antibody. Representative data from two separate experiments.

Recent data indicate that nitrogen-containing bisphosphonates induce a dose-dependent accumulation of unprenylated Rap1A in MDA-MB-231 cells [23]. We confirmed that calcium-induced modulation of cellular accumulation of ibandronate took place by demonstrating that high calcium concentration augmented ibandronate-induced inhibition of protein prenylation (Figure 8). Indeed, in both cell lines 10 μmol/l ibandronate was sufficient to produce a detectable inhibition of Rap1A prenylation in the presence of 2.0 mmol/l calcium, whereas 100 μmol/l ibandronate was required to achieve a similar effect in presence of 0.6 mmol/l calcium. Therefore, high calcium level increased the intracellular activity of ibandronate, probably by favouring cellular drug accumulation.

### High extracellular calcium concentration decreases the treatment duration required to obtain an effective ibandronate-induced cell growth inhibition

**Figure 9:**
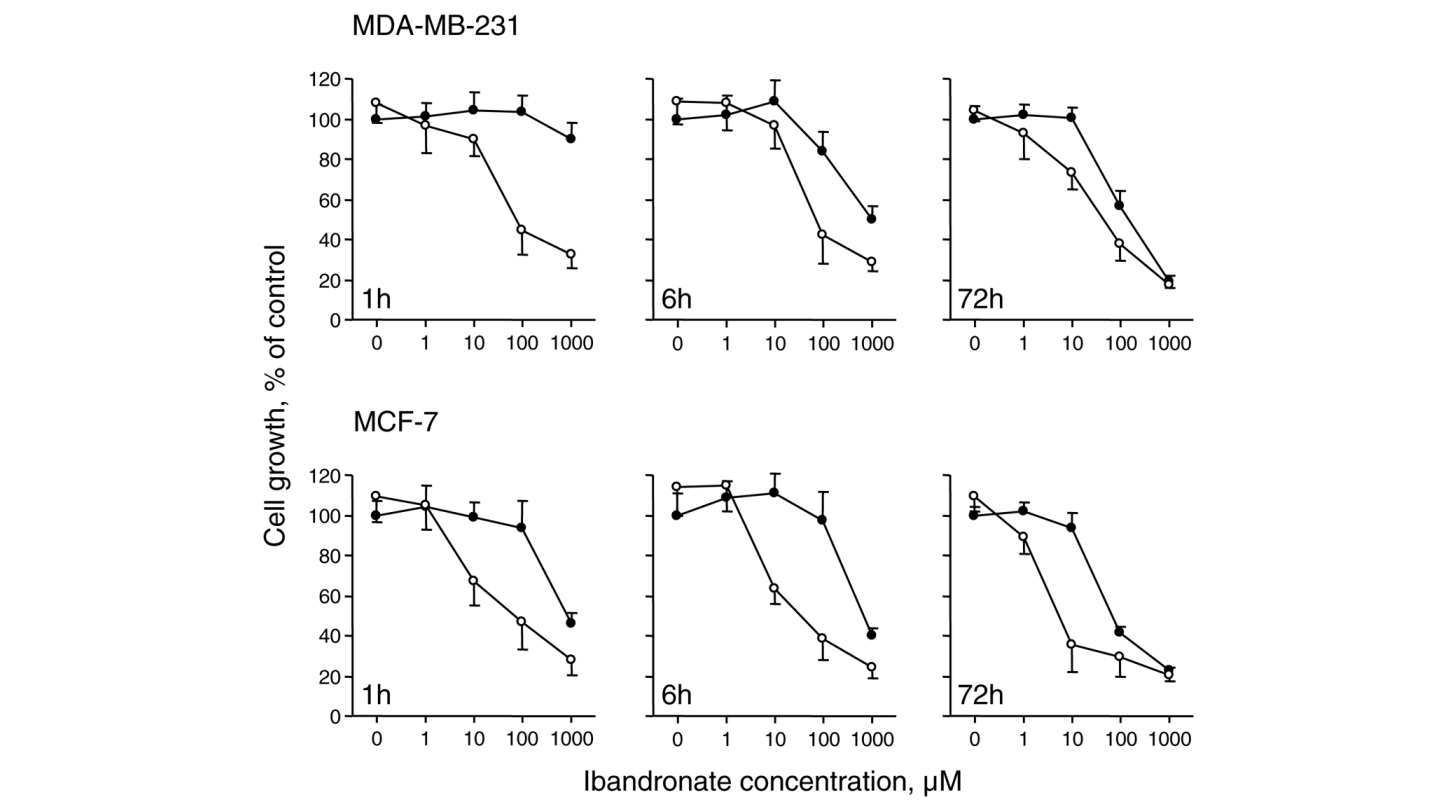
Pulse exposures of cancer cells to ibandronate in presence of low or high calcium concentrations. MDA-MB-231 and MCF-7 cells were incubated for 1, 6, or 72 hours with ibandronate (1 to 1,000 μmol/l) in culture medium containing 0.6 mmol/l calcium (filled circles) or 1.6 mmol/l calcium (open circles). Cell culture densities were evaluated at 72 hours by crystal violet staining assay. Data are presented as percentages (mean ± standard deviation) of control values, which refer to cultures with 0.6 mmol/l calcium and without ibandronate. Mean of results pooled from three experiments (*n *= 18).

Figure 9 illustrates the growth inhibitory effect of shorter exposures (1 and 6 hours) to ibandronate plus calcium at low or high concentrations. In culture medium containing 0.6 mmol/l calcium, 1 hour of ibandronate treatment had no or only a weak effect on breast cancer cell growth. As expected, continuous treatment (72 hours) increased bisphosphonate-induced inhibition of growth. In the presence of 1.6 mmol/l calcium, exposure to ibandronate for 1 hour dramatically decreased cell proliferation. Moreover, in these experimental conditions, inhibition of cell growth was more effective than after 72 hours of ibandronate treatment with low calcium concentration. Of note, 6 hours of exposure produced intermediate effects. These data brought further evidence that calcium enhances the growth inhibitory effect of ibandronate in breast cancer cells, even in the case of short treatment durations, suggesting a significant decrease in the time needed to obtain an effective cell accumulation of the bisphosphonate.

## Discussion

Metastatic bone disease is the main cause of morbidity in breast cancer patients [1]. Tumour-induced osteolysis provokes an excessive and uncontrolled release of calcium from the bone matrix, notably resulting in malignant hypercalcaemia. As a matter of fact, metastastic breast cancer cells proliferate in bone tissue microenvironment, and high calcium concentration can influence the activity of therapeutic agents aimed at treating bone metastases.

Bisphosphonates are currently used to alleviate tumour-associated bone diseases [20]. They have high affinity for calcium and accumulate in bone tissue, where they are efficiently taken up by bone-resorbing osteoclasts. On the other hand, nonresorbing cells such as tumour cells have a lesser tendency to accumulate bisphosphonates and therefore are likely to exhibit less sensitivity to these drugs [24].

In the present study, we documented the effect of ibandronate and zoledronic acid on the proliferation and the apoptosis of MDA-MB-231 and MCF-7 cells in the presence of various clinically relevant extracellular calcium concentrations (0.6 to 2.0 mmol/l). Our data show that high calcium level strongly increases the growth inhibitory effects of ibandronate and zoledronic acid in both cell lines. Moreover, these observations were not restricted to MDA-MB-231 and MCF-7 cells, because they could be extended to four other breast cancer cell lines (T-47D, ZR-75-1, Hs-578T and BT-549). Thus, the antitumour activity of bisphosphonates appears to be calcium dependent. By contrast, the cytotoxicity of the chemotherapeutic agent paclitaxel is independent of extracellular calcium. It can be inferred from these observations that calcium does not simply act by modulating cell drug sensitivity in a nonspecific manner. The chelator EGTA abrogates the influence of calcium on ibandronate-induced growth inhibition, probably by competing with the bisphosphonate for calcium complexation. Because the phosphonate groups allow effective chelation of metal ions [25], it may be speculated that the increase in extracellular complexation favours the emergence of bisphosphonate-calcium complexes, leading to changed drug pharmacokinetics at the cellular level and to a rapid enhancement of drug accumulation in tumour cells. The validity of this interpretation is confirmed by the fact that an augmentation of extracellular calcium increases the cellular accumulation of [^14^C]ibandronate, accentuates ibandronate-induced inhibition of Rap1A prenylation, and leads to effective antitumour effects of ibandronate after exposure duration of as short as 1 hour.

It is worth noting that an enhancing effect of calcium on cellular bisphosphonate accumulation and on drug activity has been demonstrated in other cell types. Indeed, a previous study showed that high extracellular calcium concentration enhances the growth inhibitory action of bisphosphonates on RAW264 macrophage-like cells [26], suggesting that the uptake of bisphosphonates by macrophages is favoured by calcium. Moreover, another study revealed that bisphosphonates are more toxic to the Caco-2 intestinal cancer cell line in the presence of calcium than in the absence of calcium [27]. As reported recently, the pyrophosphate-resembling clodronate acts as a calcium chelator and inhibits cell uptake of radiolabelled ibandronate in macrophages and osteoclasts [28]. In addition, the uptake of [^14^C]zoledronic acid or of a fluorescent analogue of alendronate by J774 macrophages and rabbit osteoclasts is enhanced by the presence of calcium and strontium, and is inhibited by addition of EGTA or clodronate [12]. That study also revealed that both EGTA and clodronate prevent bisphosphonate-induced inhibition of Rap1A prenylation in J774 cells and osteoclasts. Our results extend those observations to breast cancer cells.

From our data, it appears that calcium enhances the growth inhibitory action of zoledronic acid as well as that of ibandronate. As expected, the former bisphosphonate is more active regardless of the calcium concentration, suggesting better cell penetration or a stronger inhibition of farnesyl diphosphate synthase (a key enzyme for protein prenylation). Of note, complexes that form between different nitrogen-containing bisphosphonates and calcium are characterized by the same drug:calcium molar ratio (1:1.3) but exhibit different water solubilities [27]. This suggests that the difference in potency between bisphosphonates could be attributed partly to cellular drug pharmacokinetics.

Our observations are at variance with the findings of a recent study [23], which showed that addition of 1 mmol/l calcium partly reversed the growth inhibitory effect of zoledronic acid on MDA-MB-231 cells, whereas it enhanced that of risedronate. It must be noted, however, that in those experiments MDA-MB-231 cells were cultured in serum-free Dulbecco's modified Eagle's medium, which already contains 1.8 mmol/l calcium. Such experimental conditions are likely to obscure the actual effect of calcium increase on bisphosphonate activity.

In accordance with the findings of previous studies [4,5], MDA-MB-231 cells proved to be less sensitive to the cytotoxic action of bisphosphonates than other breast cancer cells. The activation of the p38 mitogen-activated protein kinase pathway by bisphosphonates in MDA-MB-231 cells has been associated with increased cell survival and may account for a relative resistance to these drugs [29]. In addition, MDA-MB-213 cells might also differ in their ability to take up and/or accumulate bisphosphonates. Indeed, our data show that MCF-7 cells were more prone to accumulating [^14^C]ibandronate than were MDA-MB-231 cells, suggesting that the difference in cell sensitivity to bisphosphonates may be related, at least in part, to drug pharmacokinetics. Interestingly, our data revealed that the cell sensitivity to ibandronate could be associated with the ER status of the tumour cells. This could be explained by the fact that oestrogen stimulation is critical for survival in ER-positive breast tumour cells and that, as previously reported [7], the mitogenic effects of oestrogens are completely abrogated by ibandronate. The clinical relevance of this *in vitro *observation should be further investigated to establish whether ER status could be useful in predicting cell responses to bisphosphonates.

Interestingly, unlike calcium, magnesium did not increase the growth inhibition induced by ibandronate in breast cancer cell lines. These data are in accordance with those reported in macrophages and osteoclasts, showing that addition of 1 mmol/l magnesium did not increase the internalization of a fluorescent analogue of alendronate [12]. Although the bisphosphonates have almost the same affinity for calcium and magnesium, the divalent cations may differ in their ability to coordinate with bisphosphonates and to form multinuclear complexes [25]. Indeed, magnesium demonstrates preference for mononuclear complexes, whereas calcium tends to form multinuclear species [30]. These data may account for the fact that magnesium has little impact on bisphosphonate activity.

The CaR is expressed in MDA-MB-231 and MCF-7 cells [31]. In the present study its involvement in the effects of calcium was examined by using a CaR agonist and a CaR antagonist. As the results showed, these CaR modulators had no influence on ibandronate activity. These observations further support the concept that enhanced cellular drug accumulation resulting from calcium complexation is the major mechanism that underlies calcium-induced potentiation of bisphosphonate antitumour activity.

## Conclusion

Our data indicate that extracellular calcium, at clinically relevant concentrations, increases the cytotoxicity of ibandronate by facilitating its accumulation by breast cancer cells. Therefore, calcium released during the process of bone resorption could enhance the antitumour effects of bisphosphonates and contribute to their therapeutic activity.

## Abbreviations

7-AAD = 7-amino-actinomycin; CaR = calcium-sensing receptor; EGTA = ethylene glycol tetra-acetic acid; ER = oestrogen receptor; IC_50 _= half maximal inhibitory concentration; PBS = phosphate-buffered saline; PE = phycoerythrin.

## Competing interests

FJ and JJB are recipients of a research grant from Hoffman-LaRoche (Basel, Switzerland).

## Authors' contributions

FJ designed the experiments, performed the analysis and interpreted the data, and drafted the manuscript. NK and CC carried out cell culture experiments, cell growth determination, Western blot analysis, and [^14^C]ibandronate cell accumulation. HD carried out apoptosis determination. GL discussed the results and critically revised the manuscript. JJB participated in the design of the experiments, discussed the results and revised the manuscript. All authors read and approved the final manuscript.
